# Clinical analysis of treatment strategies to cholecystocholedocholithiasis patients with previous subtotal or total gastrectomy: a retrospective cohort study

**DOI:** 10.1186/s12893-018-0388-1

**Published:** 2018-08-09

**Authors:** Mingjie Zhang, Jianxin Zhang, Xu Sun, Jie Xu, Jing Zhu, Wenbin Yuan, Qiang Yan

**Affiliations:** 1 Department of Hepatobiliary surgery, Huzhou Hospital, Zhejiang University School of Medicine (Huzhou Central Hospital), No. 198, Hongqi Road, Huzhou, 313000 Zhejiang Province China; 2 Department of General surgery, The NO.3 People’s hospital of Changxing County, No. 19, Tianneng Road, Changxing, 313104 Zhejiang Province China

**Keywords:** Cholecystocholedocholithiasis, Laparoscopic common bile duct exploration, Laparoscopic cholecystectomy, Endoscopic retrograde cholangiopancreatography, Previous gastrectomy

## Abstract

**Background:**

Previous gastrectomy can lead to an increased incidence of cholecystocholedocholithiasis (CCL) and increased morbidity rate. However, the appropriate treatment strategy for patients with CCL and a history of gastrectomy remains unclear.

**Methods:**

We performed a retrospective cohort study of patients with CCL and a history of gastrectomy who underwent either one-stage laparoscopic common bile duct (CBD) exploration with stone clearance and laparoscopic cholecystectomy (LCBDE+LC) or two-stage endoscopic retrograde cholangiopancreatography followed by LC (ERCP+LC) from May 2010 to March 2018.

**Results:**

The success rate of ERCP for CBD stone clearance was 81.2% in patients with a history of Billroth I gastrectomy and 23.7% in patients with a history of Billroth II or Roux-en-Y esophagojejunostomy [χ^2^ = 97.67, *P* < 0.001, risk ratio (RR) = 3.43]. The success rate of second-step LC after successful ERCP for removal of CBD stones and the success rate of LCBDE+LC after ERCP treatment failure were 96.8 and 87.7%, respectively, in patients with preoperative intra-abdominal adhesion evaluation scores of ≤3 points. These success rates were 28.6 and 27.6%, respectively, in patients with scores of > 3 points (χ^2^ = 59.70, *P* < 0.001, RR = 3.38 and χ^2^ = 53.41, P < 0.001, RR = 3.27, respectively).

**Conclusions:**

Based on the results of this study, ERCP+LC seems to be an attractive strategy for treatment of CCL in patients with a history of Billroth I gastrectomy, and LCBDE+LC appears to be suitable for patients with a history of Billroth II or Roux-en-Y esophagojejunostomy. Preoperative evaluation of intra-abdominal adhesions helps to reduce the conversion rate of laparoscopic surgery.

## Background

Treatment options for cholecystocholedocholithiasis (CCL) include two-stage endoscopic retrograde cholangiopancreatography (ERCP) followed by laparoscopic cholecystectomy (LC) (i.e., ERCP+LC), one-stage laparoscopic common bile duct exploration (CBDE) and LC (i.e., LCBDE+LC), and laparotomic CBDE and cholecystectomy [1, 2]. With the development of laparoscopic and endoscopic equipment and technology, open CBDE is now rarely applied in clinical practice [3]. However, whether ERCP+LC or LCBDE+LC is more beneficial in the management of patients with CCL remains unclear [4]. Patients with a history of gastrectomy have a higher incidence of CCL and morbidities requiring surgical treatment [5, 6]. The therapeutic strategy for CCL in patients with a history of gastrectomy is still being debated. This study was performed to investigate the correlative factors that influence the option of treatment modalities to allow for the development of individualized treatment strategies for patients with CCL and a history of gastrectomy.

## Methods

### Study protocol

We performed a retrospective cohort study of patients with CCL and a history of gastrectomy in Huzhou Central Hospital and the No. 3 People’s Hospital of Changxing County, China from May 2010 to March 2018. In all patients, the diagnosis of CCL was confirmed by magnetic resonance cholangiopancreatography. The study was approved by both hospitals’ ethics committees, and written informed consent was obtained from each patient. Both hospitals provide a comprehensive general surgical service and tertiary care for patients undergoing hepatobiliary surgery among a population of 3 million people. In total, 394 consecutive patients were enrolled in this study. Of these, 342 patients underwent ERCP for removal of CBD stones first, and 289 patients who were candidates for elective LC or LCBDE+LC underwent preoperative evaluation of intra-abdominal adhesions based on our previous research [7], as shown in Table 1. All ERCP procedures were performed by senior endoscopists familiar with the procedure. The patients underwent endoscopic sphincterotomy and clearance of CBD stones with a balloon or Dormia basket. Laser lithotripsy was not available. A plastic stent was introduced for patients with retained CBD stones after primary ERCP clearance. All laparoscopic operations in both departments were performed by consultant surgeons. The technique of LC or LCBDE for treatment of CCL in patients with a history of gastrectomy has been described in our previous publications [7, 8]. The clinical outcomes of each CCL management technique were documented.

**Table 1:** Evaluation score table of preoperative intraabdominal adhesions

	Score (points)	
Hyperplasia of original incision	No (0)	Yes (1)
Postoperative intestinal obstruction	No (0)	Yes (1)
Abdominal infection	No (0)	Yes (1)
Methods of gastroenterostomy	Billroth I (0)	Billroth II or Roux-en-Y (1)
Preoperative ultrasonography test	Lateral MD > 1 cm	Lateral MD < 1 cm
	Longitudinal MD > 3 cm	Longitudinal MD < 3 cm
	(0)	(2)

*MD* Movement distance

### Statistical analysis

All data were prepared and compiled using the SPSS computer program (version 19.0 for Windows; IBM Corp., Armonk, NY, USA). The chi square test and Fisher’s exact test were used for quantitative data. Step-wise regression was used for multivariate analysis to identify any confounding factors. Pearson’s correlation test was used for correlation analysis. A *p*-value < 0.05 was considered statistically significant.

## Results

### Patients’ demographic information

The clinical data of all 394 patients with CCL with a history of gastrectomy who underwent treatment from May 2010 to March 2018 were retrospectively reviewed. The patients’ ages ranged from 27 to 81 years (mean, 58 years; median, 47 years). The cohort comprised 156 (39.6%) men and 238 (60.4%) women, with a male:female ratio of 0.655:1.000. No gallbladder cancer was identified. All patients had previously undergone gastrectomy for either gastric cancer [363 patients (92.1%)] or gastroduodenal ulcers [31 patients (7.9%)]. The types of gastrectomy were distal gastrectomy in 293 patients [Billroth I gastrectomy in 103 patients (26.1%) and Billroth II gastrectomy in 190 patients (48.2%)] and total gastrectomy with Roux-en-Y esophagojejunostomy in 101 patients (25.7%), as shown in Table 2.

**Table 2:** Details of previous gastrectomy

Billroth I (*n* = 103)	Gastric antrum cancer (n = 103)		Gastroduodenal ulcer (*n* = 0)	
	Stage I	63 (61.2%)		
	Stage II	40 (38.8%)		
Billroth II (*n* = 190)	Gastric antrum cancer (*n* = 159)		Gastroduodenal ulcer (*n* = 31)	
	Stage I	9 (5.7%)	Gastric ulcer	2 (6.5%)
	Stage II	62 (39.0%)	Duodenal ulcer	29 (93.5%)
	Stage III	88 (55.3%)		
Roux-en-Y (*n* = 101)	Gastric corpus cancer (*n* = 89)		Gastric cardia cancer (*n* = 12)	
	Stage I	1 (1.1%)	Stage II	2 (16.7%)
	Stage II	46 (51.7%)	Stage III	10 (83.3%)
	Stage III	42 (47.2%)		

### ERCP management in patients with CCL and a history of gastrectomy

**Fig. 1:**
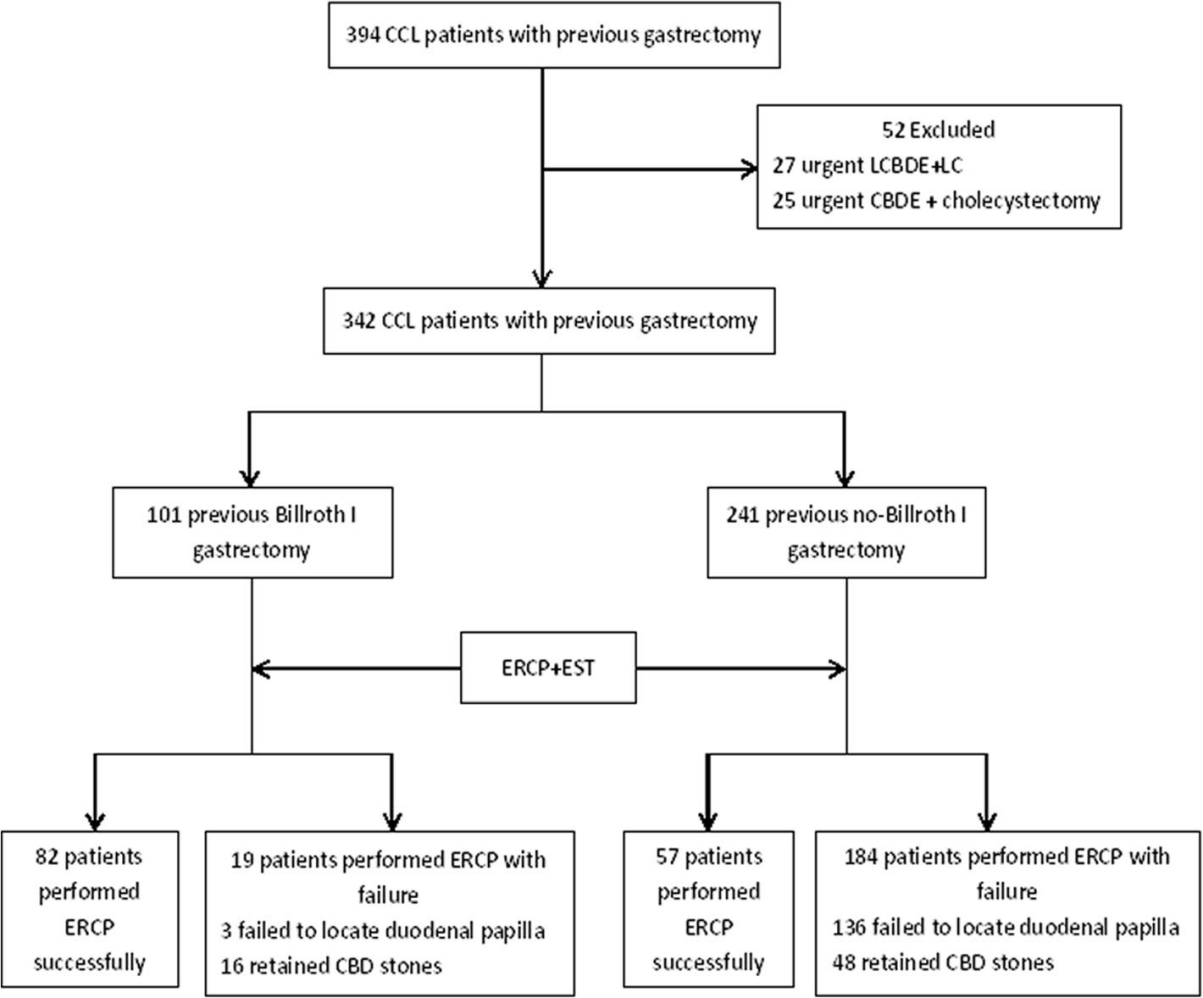
Flow chart of performing ERCP to CCL patients with previous gastrectomy. CCL: Cholecystocholedocholithiasis; LCBDE: Laparoscopic common bile duct exploration; LC: Laparoscopic cholecystectomy; CBDE: laparotomy common bile duct exploration; ERCP: Endoscopic retrograde cholangiopancreatography; EST: Endoscopic sphincterotomy

A total of 342 patients with CCL and a history of gastrectomy underwent ERCP for removal of CBD stones first. Of these, 139 patients underwent successful CBD stone clearance with ERCP, and without CBD stones retained which confirmed by postoperative magnetic resonance cholangiopancreatography confirmed that no stones had been retained. Among these 139 patients, 82 had previously undergone Billroth I gastrectomy and 57 had previously undergone Billroth II gastrectomy or Roux-en-Y esophagojejunostomy as shown in Fig. 1. The success rate of ERCP for CBD stone clearance was 81.2% in patients with a history of Billroth I gastrectomy and 23.7% in patients with a history of non-Billroth I gastrectomy [χ^2^ = 97.67, *P* < 0.001, risk ratio (RR) = 3.43], as shown in Table 3.

**Table 3:** Success rate of ERCP for CBD stones clearance in groups with different previous gastrectomy

Previous gastrectomy	Total	Success rate of ERCP	χ^2^	*P* value	RR
Billroth I	101	81.2% (82/101)	97.67	0.001	3.43^a^
Non-Billroth I	241	23.7% (57/241)			

^a^RR = Success rate of ERCP in performing Billroth I gastrectomy group / Success rate of ERCP in performing non-Billroth I gastrectomy group

*ERCP* endoscopic retrograde cholangiopancreatography, *CBD* common bile duct, *RR* relative risk

### Laparoscopic surgery in patients with CCL and a history of gastrectomy

**Fig. 2:**
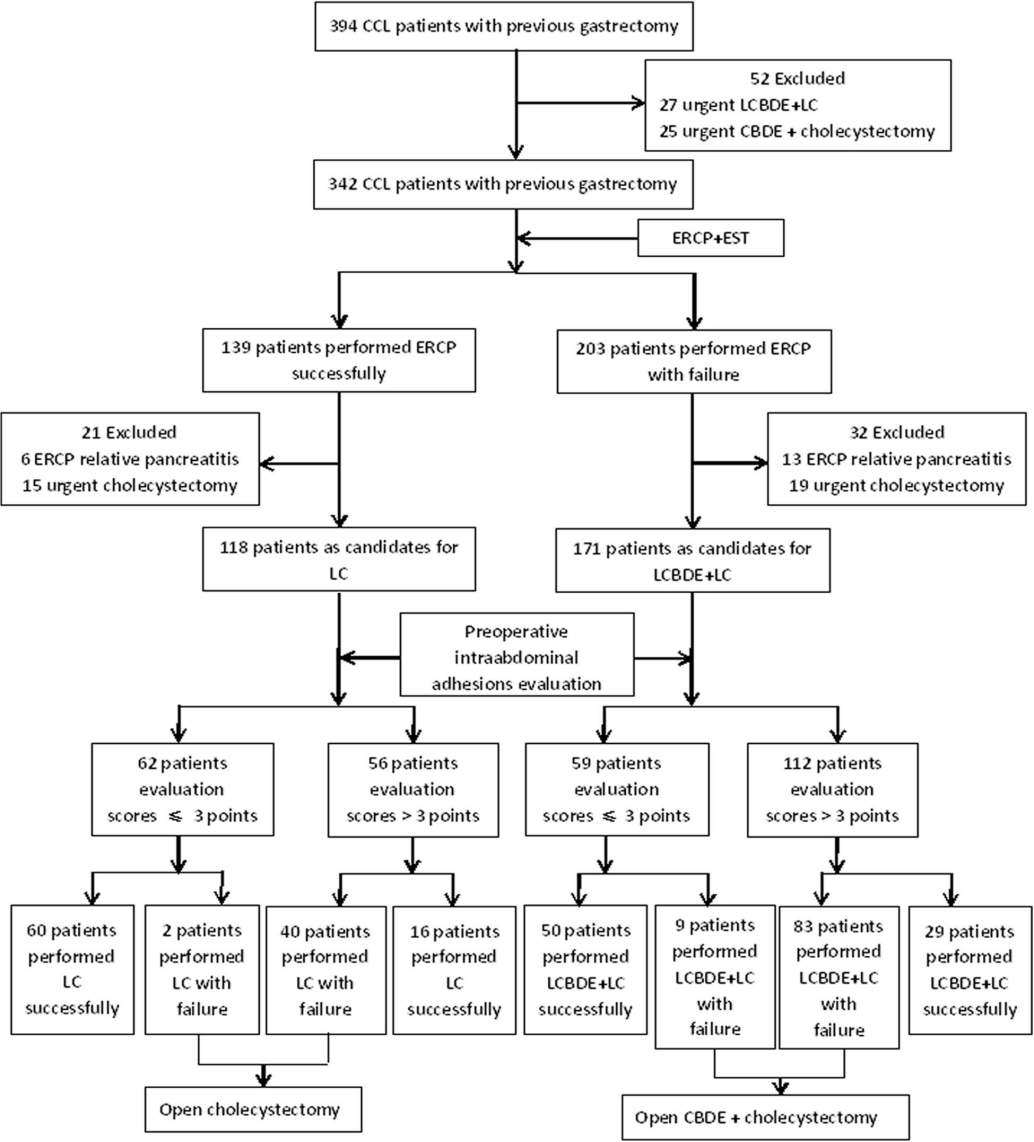
Flow chart of performing laparoscopic surgery to CCL patients with previous gastrectomy. CCL: Cholecystocholedocholithiasis; LCBDE: Laparoscopic common bile duct exploration; LC: Laparoscopic cholecystectomy; CBDE: laparotomy common bile duct exploration; ERCP: Endoscopic retrograde cholangiopancreatography

A total of 118 patients underwent LC after successful CBD stone removal by ERCP, and 171 patients underwent LCBDE+LC after ERCP treatment had failed (Fig. 2). The success rates of LC and LCBDE+LC were 96.8 and 84.7%, respectively, in patients with preoperative intra-abdominal adhesion evaluation scores of ≤3 points and 28.6 and 25.9%, respectively, in patients with scores of > 3 points (χ^2^ = 59.70, *P* < 0.001, RR = 3.38 and χ^2^ = 53.41, P < 0.001, RR = 3.27), as shown in Table 4.

**Table 4:** Success rate of laparoscopic surgery in CCL patients with previous gastrectomy

Laparoscopic surgery	Preoperative intraabdominal adhesions evaluation scores	Success rate	χ^2^	*P* value	RR
LC (*n* = 118)	≤ 3 points, *n* = 62	96.8%(60/62)	59.70	0.001	3.38^a^
	> 3 points, *n* = 56	28.6%(16/56)			
LCBDE+LC (*n* = 171)	≤ 3 points, *n* = 59	84.7%(50/59)	53.41	0.001	3.27^b^
	> 3 points, *n* = 112	25.9%(29/112)			

^a^RR = Success rate of LC in evaluation scores ≤3 points group / Success rate of LC in evaluation scores > 3 points group

^b^RR = Success rate of LCBDE+LC in evaluation scores ≤3 points group / Success rate of LCBDE+LC in evaluation scores > 3 points group

*LC* Laparoscopic cholecystectomy, *LCBDE* Laparoscopic common bile duct exploration

### Clinical outcomes

A total of 76 patients underwent ERCP+LC and 79 underwent LCBDE+LC for treatment of CCL after a previous gastrectomy. The hospital costs and length of hospital stay were greater in the ERCP+LC than LCBDE+LC group (*P* < 0.05). The incidence of postoperative bile leakage, postoperative hemorrhage, postoperative cholangitis, postoperative pancreatitis, residual CBD stones, recurrence of CBD stones, bile duct stricture, and patient death were not significantly different between the two groups (*P* > 0.05) (Table 5).

**Table 5:** Clinical outcomes of different management in CCL patients with previous gastrectomy

Clinical outcomes	ERCP+LC, *n* = 76	LCBDE+LC, *n* = 79	*P* value
Postoperative bile leakage	4(5.3%)	5(6.3%)	0.777
Postoperative hemorrhage	2(2.7%)	4(5.1%)	0.433
Postoperative cholangitis	10(13.2%)	6(7.6%)	0.255
Postoperative pancreatitis	8(10.5%)	10(12.7%)	0.679
Residual CBD stones	6(7.9%)	4(5.1%)	0.473
CBD stones recurrence^a^	5(6.6%)	7(8.9%)	0.595
Bile duct stricture^a^	0	0	1.0
Hospital costs (RMB)	37,652 ± 112.3	23,162 ± 89.6	0.032*
Hospital stay (days)	14.7 ± 1.8	6.5 ± 1.5	0.013*
Death	0	0	1.0

*RMB* (Renminbi) Currency unit of China, *ERCP* endoscopic retrograde cholangiopancreatography, *LC* Laparoscopic cholecystectomy, *LCBDE* Laparoscopic common bile duct exploration, *CBD* common bile duct

^a^The median follow-up time was 37 (range 1–93) months

*p < 0.05

## Discussion

It is widely accepted that patients with a history of gastrectomy have an increased incidence of CCL and increased morbidities requiring surgery [9]. The exact mechanisms for these observations remain unclear. According to the literature, the complex interaction between sectioning of the nerve supply to the gallbladder and the change in cholecystokinin secretion plays an important role [10, 11]. In gastric operations, reconstruction of the digestive tract may decrease passage of food through the duodenum, which probably decreases cholecystokinin secretion and reduces gallbladder motility, facilitating gallstone formation [12]. In addition, the hepatic branch of the vagus nerve is unavoidably damaged during surgical operations for gastric cancer because of the need for extended lymphadenectomy; moreover, the absence of or damage to the hepatic branch may cause dysregulation of gallbladder emptying, which may in turn contribute to gallstone formation [13]. Therefore, individualized and appropriate treatment strategies for CCL in patients with a history of gastrectomy are very valuable because such patients are often encountered in clinical practice.

Historically, the treatment of CCL required open laparotomy and CBDE [14]. After the introduction of ERCP and endoscopic sphincterotomy in the 1970s, ERCP+LC provided a less invasive option for treating CCL and has largely replaced CBDE in the management of CCL in the last two decades [15, 16]. With the development of laparoscopic equipment and technology, LCBDE has been widely used in clinical practice since its first introduction in 1991 [17–19]. In contrast to ERCP+LC, which is generally performed in two stages, LCBDE+LC for treatment of CCL is generally performed in a single stage. Additionally, this procedure appears to have a shorter hospital stay and similar stone clearance rate, relative cost-effectiveness while preserving the function of the sphincter of Oddi, and fewer ERCP-related complications [20, 21]. Although many studies have proven that LCBDE+LC is both feasible and effective in the management of CCL [22, 23], one retrospective cohort study performed in the United States showed that the overall use of ERCP+LC for treatment of CCL increased from 52.8% of admissions in 1998 to 85.7% in 2013 and that the percentage of patients with CCL undergoing CBDE (including open CBDE and laparoscopic CBDE) decreased from 39.8 to 8.5% in the same period. These results indicate that despite the potential benefits of LCBDE+LC over ERCP+LC for managing CCL, the current trends in CCL management continue, and CBDE may be at risk of disappearing from the surgical armamentarium [24]. Although the results of various studies strongly support this view, which treatment strategy is more beneficial to patients with CCL, especially those with a history of gastrectomy, still needs further investigation.

Most surgical specialists believe that either ERCP+LC or LCBDE+LC should be specifically chosen to treat patients with CCL in clinical practice based on the size and quantity of CBD stones, whether the CBD stones are combined with gallstones, the location and severity of the obstruction, and especially the level of the surgeon’s experience in ERCP or LCBDE at individual treatment centers [25, 26]. In the two hospitals of the present study, the ERCP technique was introduced in 2001 and has been applied to clinical practice for almost 20 years. Our endoscopists have accumulated abundant experience in performing ERCP to treat CCL, even in patients with a history of gastrectomy, and ERCP is typically the first-line treatment for CCL in both hospitals. We reviewed the clinical data of patients with CCL who underwent ERCP from 2001 to 2018 in our two medical centers and found that a history of gastrectomy was the most common cause of ERCP treatment failure (38.8%), followed by compact CBD stones (21.3%) and duodenal papilla hemorrhage (14.2%). However, the success rate of ERCP in patients with CCL with a history of gastrectomy still reached 67.9%. Previous randomized trials and meta-analyses have demonstrated the safety and efficacy of ERCP management for CCL with a success rate of 61.7 to 94.6% [27, 28]. Compared with our observation, the success rate of ERCP in patients with CCL and a history of gastrectomy is in accordance with the average level. These results indicate that a history of gastrectomy may be an important reason for failure of ERCP, but not a contraindication. Identification of the risk factors for ERCP failure in patients with CCL and a history of gastrectomy is important and was the major aim of our study. We selected gastroenteric anastomosis as the candidate risk factor and conducted a retrospective cohort study from May 2010 to March 2018, and we found that patients with a history of Billroth I gastrectomy have a higher success rate of ERCP for clearance of CBD stones and that ERCP might therefore be the first choice to treat choledocholithiasis in these patients.

Regardless of whether ERCP is performed successfully, all patients with CCL will inevitably undergo second-step LC or one-stage CBDE+cholecystectomy [28]. Previous upper abdominal surgery, especially gastrectomy, is a relative contraindication for laparoscopic surgery [29]. In one study, all surgical failures were attributable to adhesions, which included adhesions to the anterior abdominal wall at the site of insertion of the initial trocar and adhesions around the gallbladder and CBD [30]. In our two hospitals, LC and LCBDE were first introduced in 1996 and 2008, respectively, and gained widespread clinical acceptance even in patients with a history of upper abdominal surgery. We adopted a preoperative intra-abdominal adhesion evaluation procedure in 2011 to anticipate the severity of intra-abdominal adhesions, and this evaluation procedure significantly reduced the conversion rate of LC in these patients as shown in our previous research [7, 8]. In the present study, we chose the preoperative intra-abdominal adhesion evaluation score as a risk factor for conversion of LCBDE or performance of second-step LC in patients with CCL and a history of gastrectomy. We found that the success rates of laparoscopic surgery in patients with CCL and a history of gastrectomy are different when the preoperative intra-abdominal adhesion evaluation scores vary. When the evaluation score is > 3 points in an individual patient, extensive intra-abdominal adhesions are suspected or present, and safe peritoneal access is therefore needed. Open laparoscopy is the most recommended method in these patients with Hasson cannula [31, 32]. The peritoneal access technique is not difficult, but it is essential to increase the success rate of initial trocar insertion. Therefore, comprehensive and accurate preoperative evaluation of the severity of adhesions is important, and application of this procedure to clinical treatment would help to reduce the conversion rate of laparoscopic surgery in these patients.

At last, we collected the clinical information of the patients who were performed ERCP+LC or LCBDE+LC successfully, and we found that the postoperative complications have no differences between two groups, but the hospital costs and length of hospital stay were reduced in LCBDE+LC group. These results accord with the previous research conclusions.

Although majority of carried out studies confirmed that the incidence of CCL after gastric resection is increased compared with the people without gastrectomy history, but performing prophylactic cholecystectomy during gastric cancer surgery is still being debated [33]. In the fact, according to the data from the available published literature, the incidence of gallstone formation and symptomatic cholecystolithiasis requiring cholecystectomy after gastrectomy is low [34]. Based on these observations, we believed that routine prophylactic cholecystectomy may not be necessary for all patients undergoing gastrectomy, but identify the risk factors which contribute to gallstone formation and subsequent cholecystectomy is really mattered and which can help surgeons to make their rational surgical treatment strategies and avoid subsequent surgery or surgical overtreatment.

Limitations and possible biases in this study are the lack of randomization, which may have caused some selection bias, and the small number of patients, making the detection of small differences between the study groups unreliable.

## Conclusions

This study has shown that ERCP+LC seems to be an attractive strategy for treatment of CCL in patients with a history of Billroth I gastrectomy and that LCBDE+LC is suitable for patients with a history of Billroth II or Roux-en-Y esophagojejunostomy. Introduction of preoperative evaluation of intra-abdominal adhesions is beneficial for reducing the conversion rate of laparoscopic surgery in patients with CCL and a history of gastrectomy.

## Abbreviations

CBD: Common bile duct
CCL: Cholecystocholedocholithiasis
ERCP: Endoscopic retrograde cholangiopancreatography
EST: Endoscopic sphincterotomy
LC: Laparoscopic cholecystectomy
LCBDE: Laparoscopic common bile duct exploration
MRCP: Magnetic resonance cholangiopancreatography
RR: Relative risk
